# Strategies for Reducing or Preventing the Generation of Oxidative Stress

**DOI:** 10.1155/2011/194586

**Published:** 2011-12-10

**Authors:** B. Poljsak

**Affiliations:** Laboratory for Oxidative Stress Research, Faculty of Health Sciences, University of Ljubljana, Zdravstvena Pot 5, 1000 Ljubljana, Slovenia

## Abstract

The reduction of oxidative stress could be achieved in three levels: by lowering exposure to environmental pollutants with oxidizing properties, by increasing levels of endogenous and exogenous antioxidants, or by lowering the generation of oxidative stress by stabilizing mitochondrial energy production and efficiency. Endogenous oxidative stress could be influenced in two ways: by prevention of ROS formation or by quenching of ROS with antioxidants. However, the results of epidemiological studies where people were treated with synthetic antioxidants are inconclusive and contradictory. Recent evidence suggests that antioxidant supplements (although highly recommended by the pharmaceutical industry and taken by many individuals) do not offer sufficient protection against oxidative stress, oxidative damage or increase the lifespan. The key to the future success of decreasing oxidative-stress-induced damage should thus be the suppression of oxidative damage without disrupting the wellintegrated antioxidant defense network. Approach to neutralize free radicals with antioxidants should be changed into prevention of free radical formation. Thus, this paper addresses oxidative stress and strategies to reduce it with the focus on nutritional and psychosocial interventions of oxidative stress prevention, that is, methods to stabilize mitochondria structure and energy efficiency, or approaches which would increase endogenous antioxidative protection and repair systems.

## 1. Introduction

High levels of reactive oxygen species (ROS), compared to antioxidant defenses, are considered to play a major role in diverse chronic age-related diseases and aging. The cells of all present aerobic organisms produce the majority of chemical energy by consuming oxygen in their mitochondria. Mitochondria are thus the main site of intracellular oxygen consumption and the main source of ROS formation [1–3]. Mitochondrial ROS sources are represented by the electron transport chain and the nitric oxide synthase reaction. Nonmitochondrial sources of ROS include environmental pollutants, pollutants in food, radiation, or they are the by-products of other metabolic processes in organisms. Majority of free radicals are generated inside the cell rather than coming from the environment [1, 4, 5]. Sources of O_2_^−∙^, the one electron reduction product of molecular oxygen, include NAD(P)H oxidases (NOX), xanthine oxidase (XO), mitochondria, and uncoupled nitric oxide synthase (NOS). Additionally, peroxynitrite and nitroxidative stress have also been implicated in various aspects of nitrooxidative cellular damage since peroxynitrite also yields secondary one-electron oxidants [6]. Cellular and tissue defenses against ROS include the enzymes superoxide dismutase (Mn-SOD, Cu/Zn-SOD, and extracellular (EC)-SOD), catalase, glutathione peroxidase, peroxiredoxins and the nonenzymatic antioxidants, glutathione (GSH), thioredoxin, ascorbate, *α*-tocopherol, and uric acid [7]. In reality, the oxidative damage potential is greater than antioxidant defence and thus there is a constant small amount of toxic-free radical formation, which escapes the defense of the cell. Estimates of how much oxygen reacts directly to generate free radicals vary; however, typically cited values are around 1.5–5% of the total consumed oxygen [8, 9]. These estimates have been questioned by Hansford et al. [10] and Staniek and Nohl [11], who suggested that H_2_O_2_ production rates were less than 1% of consumed O_2_. Yet, even if we accept a conservative value of 0.15%, this still represents a substantial amount of free radicals formation [12]. The rate of generation of H_2_O_2_ is dependent on the state of mitochondria as determined by the concentration of ADP, substrates, and oxygen [13]. It was observed that maximum lifespan correlates negatively with antioxidant enzyme levels and free-radical production, and positively with the rate of DNA repair [2]. While data from studies in invertebrates (e.g., C. elegans and Drosophila) and rodents show a correlation between increased lifespan and resistance to oxidative stress (and in some cases reduced oxidative damage to macromolecules), direct evidence showing that alterations in oxidative damage/stress play a role in aging are limited to a few studies with transgenic Drosophila that overexpress antioxidant enzymes [14]. Invertebrate and vertebrate modes should be separated, as the data and results are quite disparate when estimating the role of antioxidants on longevity. According to de Grey ([15] and references therein), “a series of studies over many years has conclusively disproved the hypothesis that longevity in warm-blooded animals (homeotherms) correlates with high levels of antioxidants: in fact, these variables generally exhibit a strong negative cross-species correlation.” It is possible that the antioxidants do not reach the sites of free-radical generation, especially when mitochondria are the primary source of ROS. 

On the other hand, adding more SOD and catalase enzymes in genetic experiments has extended the lifespan of model organisms, indicating that catalytic antioxidants might have the ability to extend lifespan [16] although recent studies found that only one (the deletion of the Sod1 gene) of the 18 genetic manipulations studied had an effect on the lifespan of mice [17]. The oxidative stress theory of aging predicts that loss of SOD activity should res ult in increased sensitivity to oxidative stress, since the organism would be less able to detoxify ROS. On the other hand, CuZnSOD activity increment might accelerate hydrogen peroxide formation and might promote oxidative damage. Overexpression of the major antioxidant enzymes (CuZnSOD, catalase, or combinations of either CuZnSOD and catalase or CuZnSOD and MnSOD) is insufficient to extend lifespan in transgenic mice [17, 18]. In the transgenic mice which express human CuZnSOD gene, an oversupply of this enzyme is not beneficial to the brain and causes increased thiobarbituric-reactive substances (TBARS), an index of lipid peroxidation [19]. Additionally, mice deficient in both Mn superoxide dismutase and glutathione peroxidase-1 have an increased oxidative damage and a greater incidence of pathology but no reduction in longevity [20]. Similarly, single or multiple deletions of various SOD isoforms in *Caenorhabditis elegans* did not shorten lifespan despite protecting against oxidative stress [21–23]. In fact, loss of SOD-2 both increased lifespan at the same time as it increased sensitivity to oxidative stress in this model organism [24]. It seems that greater oxidative damage has no impact on the age-related mortality profiles of the animals in question [25]. Besides, genetic approaches currently have severe limitations in humans and considering the fact that the catalytic enzymes themselves would be degraded in the digestive tract, the rationale solution would be to search for SOD and catalase mimetics [18] (for more information see also paragraph on mimetics).

Also the currently available and consumed antioxidants (e.g., vitamin C, E, and beta-carotene) show inefficacy in ameliorating oxidative stress-mediated diseases. The use of multivitamins/minerals (MVMs) has grown rapidly over the past several decades, and dietary supplements are now used by more than half of the adult population in the United States. On the other hand, some recent studies showed that antioxidant therapy has no effect and can even increase mortality [26–37]. Ristow and coworkers showed that nutritive antioxidants completely abolish the extension of lifespan by inhibiting a process called mitohormesis [38]. Many clinical trials in which individuals received one or more synthetic antioxidants failed to obtain beneficial results. Results of clinical trials on exogenous antioxidants intake are thus conflicting and contradictory. There are evidently homeostatic mechanisms in cells that govern the amount of allowable antioxidant activity. This indicates that other substances in fruits and vegetables, or a complex mix of substances (e.g., inhibitors of P450 (grapefruit, garlic), inhibitors of cell proliferation (resveratrol, green tea polyphenols, and curcumin), antagonists of estrogen (flavonoids), inhibitors of metastases (flavonoids), and inhibitors of angiogenesis (genistein, epigallocatechin galate), may contribute to the better cardiovascular health and decreased cancer incidence observed in individuals who consume more of fruits and vegetables [39, 40]. There is also the problem regarding the dosing of synthetic antioxidants; for example there are claims that RDA levels of vitamins C and E are too low to prevent oxidative stress. Besides, a typical oxidative stress status of an individual is not established yet and is difficult to measure [41]. The intake of only one antioxidant could alter the complex system of endogenous antioxidative defense of cells, or alter the necrosis or apoptosis pathways. Changing the level of one antioxidant causes a compensatory change in others, while the overall antioxidant capacity remains unaffected. Dosing cells with exogenous antioxidants might decrease the rate of synthesis or the uptake of endogenous antioxidants, so that the total “cell antioxidant potential” remains unaltered. Cutler [42, 43] described “the oxidative stress compensation model” which explains why dietary supplements of antioxidants have minimum effect on longevity. Cutler explains that most humans are able to maintain their set point of oxidative stress and so no matter how much additional antioxidant supplements they consumed in their diet, further decrease in oxidative stress does not occur. Antioxidant supplements thus do not appear in significantly decrease oxidative stress or/and increase life expectancy in humans [44]. For example, several scavengers and neutralizers of peroxynitrite exist, such as peroxiredoxins, metalloporphyrins, simple thiol-based antioxidants (such as mercaptoalkylguanidines, *N*-acetylcysteine, and dihydrolipoic acid), ebselen, certain selenium-containing proteins (selenoprotein P, glutathione peroxidase), tempol, cabergoline, acetaminophen as well as drugs with demonstrated *in vitro *peroxynitrite-scavenging effects (hydralazine, pindolol, zileuton, penicillamine, simvastatin, edaravone, propofol, deprenyl, rasagiline, and 1,4-dihydropyridine calcium antagonists), and numerous molecules that are present in natural or dietary products (carotenoids, polyphenol oligomers, and epicatechin), but their *in vivo* and so their therapeutic potential as peroxynitrite neutralizing agent is low (for review see [45]).

We have to realize that the use of synthetic antioxidants is not an alternative to regular consumption of fruits and vegetables. It is probable that many antioxidants are still undiscovered. Furthermore, the combination of antioxidants in fruits and vegetables causes their reciprocal regeneration and intensifies their defense against free radicals. Additionally, complete rescue of the exogenous antioxidants regarding oxidative stress does not occur because specific antioxidants cannot penetrate the blood-brain barrier, are poor absorbed (problematic membrane transport), and are converting to prooxidants (e.g., ascorbates and carotenoids) under certain physiological conditions [46]. Thus, it is important where in the cell a specific antioxidant quenches the free radicals, since different cells of the human body present large differences regarding proliferative activity, apoptotic rate, ROS production, and DNA repair capability. Antioxidants that are reducing agents can also act as prooxidants, since they are capable of reacting with molecular oxygen (e.g., ascorbic acid), and generate superoxide radicals under aerobic conditions [46]. These will dismutase to H_2_O_2_ that can enter cells and react with superoxide or reduced metal ions to form highly damaging hydroxyl radicals [47]. The presence of redox cycling metal ions with antioxidants might result in a synergistic effect, resulting in an increased free radical formation (see also Section 2.5). Whether an antioxidant functions as an antioxidant or prooxidant is determined by at least 3 factors: (1) the redox potential of the cellular environment; (2) the presence/absence of transition metals; (3) the local concentrations of that antioxidant [48].

Additionally, the beneficial physiological cellular use of ROS is now being demonstrated in different fields, including intracellular signaling and redox regulation. It is well documented that relatively low levels of ROS act physiologically at the signaling level, modulating proliferation [49], apoptosis [50], and gene expression through the activation of transcription factors [51] such as NF-kappa-B and hypoxia-inducible-factor-1*α* (HIF) [52]. Known inducers of NF-kappa-B activity are highly variable and besides ROS include also tumor necrosis factor alpha (TNF*α*) and interleukin-1-beta (IL-1*β*) [53, 54]. ROS have been recognized to act as signaling intermediates for cytokines, including IL-1 and TNF*α* [55–57]. Proinflammatory cytokines, tumor necrosis factor (TNF)-*α*, interleukin-1*β* (IL-1*β*), and interferon-*γ* (IFN-*γ*), can additionally increase oxidative stress in humans [58], inducing production of reactive oxygen species (ROS), which have been suggested to act as second messengers [59]. Increased oxidative stress, on the other hand, can result in the up-regulation of antioxidant defense proteins, metallothioneins, and heat shock proteins, among others.

Thus, our cells also generate some hydrogen peroxide deliberately for use as a chemical signal that regulates everything from glucose metabolism to cellular growth and proliferation [60]. Reactive oxygen species in moderate concentrations are also essential mediators of defense against unwanted cells. If administration of antioxidant supplements decreases free radicals, it may interfere with essential defensive mechanisms for ridding the organism of damaged cells, including those that are precancerous and cancerous [61]. Thus, antioxidant supplements may actually cause some harm [27, 28, 62–64]. Our diets typically contain safe levels of vitamins, but high-level antioxidant supplements could potentially upset an important physiological balance [27, 28, 62–64].

**Is Consumption of Fruit and Vegetable Better than Intake of Synthetic Antioxidants?**

People who eat fruits and vegetables, which happen to be good sources of antioxidants and other phytochemicals, have a lower risk of heart disease and some neurological diseases [65], and there is evidence that some types of vegetables and fruits in general, protect against a number of cancers [66] as epidemiological studies revealed, without providing the answer whether any specific bioactive molecules within fruit and vegetable have a special contribution on lower incidence. However, 80% of American children and adolescents and 68% of adults do not eat five portions a day [67]. Inadequate dietary intakes of vitamins and minerals are widespread, most likely due to excessive consumption of energy-rich, micronutrient-poor, and refined food [68]. Even in countries that in the past had high consumption of fruits and vegetables, their consumption has been lowering [69]. The impoverishment of the soil (resulting from the abnormal exploitation of the soil itself, acid rain, increasing desertification, pollution, etc.), the often uncontrolled use of pesticides, the processes of refinement of vegetables, and the processes of transformation, storage and even cooking of foods, can affect the antioxidant content of fruits and vegetables. Today, fruits and vegetables are depleted of some essential micronutrients and antioxidants because of the intensified type of production or because of postharvest processes, transport and storage [70].

## 2. Is There Any Alternative?

Many people consume synthetic antioxidants in order to decrease oxidative stress, to modulate the aging process, and to extend the health-span. In the USA, supplements represent a market of over $7 billion/year [71] and exceed $30 billion worldwide [72]. Some alternatives will be discussed which might be effective in decreasing oxidative stress as well as cheaper to implement in daily life. The key to the future success of decreasing oxidative-stress-induced damage should be the suppression of oxidative damage without disruption of the well-integrated antioxidant defense network. The approach to neutralize free radicals with antioxidants should be changed into prevention of free radical formation in the first place. Reducing the free radical formation should be more efficient than trying to neutralize free radicals after they have been produced. Recent evidence emerging from different studies discussed below shows that such possibilities exist. In the following section, we describe different processes oriented in decreasing the oxidative stress of an organism, mainly by methods which would keep the respiratory chain in cells working optimal without an increased ROS formation or methods which increase defence and repair systems. Or as Villeponteau [73] would explain “stabilizing mitochondria structure and energy efficiency is an obvious target for decreasing oxidative free radicals.”

### 2.1. The Induction of Adaptive Responses to Stress Conditions: The Role of Hormesis Effect as an Example of a Beneficial Type of Stress

The term “hormesis” describes beneficial actions resulting from the response of an organism to a low-intensity stressor. The basic concept behind the idea of hormesis is to provoke the intrinsic capability of a body rather than to supply exogenous natural or synthetic antioxidants to try to compensate for age-related decline of physiological activities in the overall maintenance mechanisms of life [74]. Hormesis might act through conserved mechanisms of its induction. Hormetic pathways, activated by phytochemicals, may involve kinases and transcription factors that induce the expression of genes that encode antioxidant enzymes, protein chaperones, phase-2 enzymes, neurotrophic factors, and other cytoprotective proteins. Specific examples of such pathways include the sirtuin-FOXO pathway, the NF-kappaB pathway, and the Nrf-2/ARE pathway [75]. For example, activation of the Nrf-2-ARE pathway by sulforaphane and curcumin was observed [76–78]. More than 60 other molecules were described that induce the Nrf2 network, most of them found in our daily diet. Although known as typical antioxidants, a closer look reveals that these molecules induce an initial mitochondrial or cytosolic ROS formation and thereby trigger an adaptive stress response and hormesis, respectively [79]. From an evolutionary perspective, the noxious properties of such phytochemicals play an important role in dissuading insects and other pests from eating the plants. However, at the subtoxic doses ingested by humans that consume the plants, the phytochemicals induce mild cellular stress responses [75]. Other examples of moderate (usually intermittent) stress inducers include ischemic preconditioning, exercise and dietary energy restriction [80]. Also electrophilic chemicals (e.g., BHT) upregulate the antioxidant-electrophile response element [81, 82]. This probably occurs through an alteration in the redox state of the target cells which causes activation of protein kinases, the activation of the Nrf2 transcription factor, and the upregulation of phase II and antioxidant enzymes, similar to responses that occur under mild chemical stress [83]. As a result, cells increase their production of cytoprotective and restorative proteins.

Finkel and Holbrook [84] stated that the best strategy to enhance endogenous antioxidant levels may actually be oxidative stress itself, based on the classical physiological concept of hormesis. The induction of both specific and general stress responses increases cellular resistance to subsequent more severe stress. Early responses result in the post-translational activation of preexisting defenses as well as activation of signal transduction pathways that initiate late responses, namely, the *de novo* synthesis of stress proteins and antioxidant defenses [85]. Studies have reported the antiaging and life-prolonging effects of a wide variety of so-called stressors, such as prooxidants, aldehydes, caloric restriction, irradiation, UV radiation, heat shock, and hypergravity. Heat and cold can also work as hormesis agents [86, 87]. Thermal stress induces a heat-shock response involving increased expression of heat-shock proteins (chaperonins). These lead to protection against heat-induced molecular damage, particularly partial denaturation of proteins, by promoting the restoration of protein function via molecular chaperone activity. Overexpression of certain HSPs in the mitochondria can significantly extend the longevity of normal-lived animals [88, 89].

### 2.2. Caloric Restriction

Caloric restriction (CR) can also work as a hormesis agent. A growing body of evidence supports the hypothesis that CR, with no malnutrition and an adequate mineral intake, also works by decreasing oxidative stress [90, 91]. The hormesis hypothesis of caloric restriction [47, 92, 93] states that the underlying mechanism of dietary restriction is the activation of a defense response that evolved to help organisms to cope with the adverse conditions. The theory unites previously disparate observations about ROS defenses, apoptosis, metabolic changes, stress resistance, and hormonal changes [94]. The work of Lee and Yu [95], Koizumi et al. [96], and Chen and Lowry [97] strongly suggest that food restriction (energetic stress) enhances the overall antioxidant capacity to maintain the optimal status of intracellular environments through the concerted interactions of cellular components to regulate ROS, and membrane stability against peroxidative stress [95]. However, recent studies do not provide a compelling evidence that the CR-associated longevity is antioxidant mediated [98]. Attention is thus directed to other targets of nutrient signaling. Four pathways have been implicated in mediating the CR effect. These are the insulin-like growth factor (IGF-1)/insulin signaling pathway, the sirtuin pathway, the adenosine-monophosphate-(AMP-) activated protein kinase (AMPK) pathway, and the target of rapamycin (TOR) pathway [98]. The results of various studies do not support the view that induction of antioxidant enzymes occur in CR animals. Although CR animals produce fewer free radicals, their metabolic rate (oxygen consumption per gram of tissue) is not reduced. A main causal factor pertaining to the reduction of oxidative stress in animals subjected to continued CR relates to a decrease in mitochondrial free radical generation. CR promotes a metabolic shift resulting in more efficient electron transport in the mitochondrial respiratory chain [90]. Faster and more efficient electron transport may lead to a lower production of ROS by mitochondria. This occurs because of reduced leakage of electrons from the respiratory chain and/or lower oxygen concentrations in the mitochondrial microenvironment [99, 100]. It was reported that long-term CR led to a 45% decrease in the rate of mitochondrial H_2_O_2_ generation and 30% decrease in oxidative damage to mtDNA in the rat heart [91], and a 28% reduction in the rate of mitochondrial ROS generation and 30% decrease in oxidative damage to mtDNA in rat skeletal muscle [101]. Many of the current theories on dietary restriction can be united in the hormesis hypothesis.

The so-called adaptive response processes may explain how ROS formation could culminate in promotion of health and life span. Increased ROS induce endogenous defence mechanisms culminating in increased stress resistance. Interestingly, low doses of ROS with intermittent duration seem to exert such effects, whereas higher doses of ROS are unquestionably detrimental [98]. Increased oxidative stress over an extended period of time is harmful as well as increased “antioxidative stress.”

Data from epidemiologic studies suggest that calorie restriction can have beneficial effects on the factors involved in the pathogenesis of primary and secondary aging and life expectancy in humans [102].

#### 2.2.1. Calorie Restriction Mimetics, Xenohormesis, and Mitohormesis

Since long-term CR is extremely difficult to maintain, the solution could be provided by CR mimetics. Calorie restriction mimetics are agents or strategies that can mimic the beneficial health-promoting and antiaging effects of CR without having to actually radically restrict diet [103]. Several compounds have been proposed as potential calorie-restriction mimetics, such as plant-derived polyphenolic molecules (e.g., quercetin, butein, and piceatannol), insulin-action enhancers (e.g., metformin), or pharmacological agents that inhibit glycolysis (e.g., 2-deoxyglucose) [104]. Promising candidates for calorie restriction mimetics are those that intersect with the critical signaling pathways identified above and include biguanides such as metformin that target the insulin signaling pathway, stilbenes (e.g., resveratrol) that affect sirtuin activity and improve mitochondrial function and energy balance [105–107], and drugs such as rapamycin that interact with mTOR signaling [108].

Resveratrol, which is present in grapes, peanuts, and several other plants, is a potent inducer of the sirtuin/Sir2 family of NAD1-dependent deacetylases. According to Oberdoerffer, Sirtuins are behind the putative effect of calorie restriction on longevity [109]. *SIRT1*, one of the 7 mammalian sirtuin genes, regulates several biological functions, including cell survival, which has led to the theory that sirtuins mediate some of the effects of calorie restriction in mammals [106, 110]. Resveratrol has been reported to activate Sir2/SIRT1 and extend the lifespan of yeast [111], nematode worms, fruit flies [112], and mice consuming a high caloric diet [106], but not in normal mice [113]. However, the effect of resveratrol on lifespan in *C. elegans* and *Drosophila* was reinvestigated by Bass et al. [114]. Authors conclude that previously reported lifespan increases were in fact due to natural variability in C. *elegans *lifespans [114]. Similarly, recent research of Burnett et al. [115] re-examined the reported effects of sirtuin overexpression on ageing and found that standardization of genetic background and the use of appropriate controls abolished the apparent effects in both *C. elegans* and *Drosophila*. Authors found that dietary restriction increased fly lifespan independently of *dSir2*. Kaeberlein and Powers III [116] and Kaeberlein et al. [117] believe that the Sir2 gene has no relevance to CR. They have proposed that the caloric restriction increases lifespan by decreasing the activity of the Target of Rapamycin (TOR) kinase. CR deactivates TOR pathway, thus slowing aging and delaying diseases of aging [118].

Additionally, pharmacological strategies to prevent tyrosine nitration which is the proof of principle of the nitrated protein-mediated damage hypothesis could include SOD-mimics and peroxynitrite decomposition catalysts, for example, manganese porphyrins [45]. Approaches to inhibit protein tyrosine nitration and subsequent toxicity by the use of cell permeable tyrosine-containing peptides were reported [119]. Tyrosine-containing peptides should protect by scavenging peroxynitrite-derived radicals and not by direct reactions with peroxynitrite as they neither increase the rate of peroxynitrite decomposition nor decrease the bimolecular peroxynitrite-mediated oxidation of thiols [119]. A variety of manganese-containing coordination compounds, frequently termed superoxide dismutase (SOD) mimics, have been reported as well to have SOD activity in vitro and to be effective at improving conditions related to increased oxidative stress in multicellular organisms [120]. Besides, metalloporphyrins, as well as Mn cyclic polyamines, Mn salen derivatives, and nitroxides were all originally developed as SOD mimics. The same thermodynamic and electrostatic properties that make them potent SOD mimics may allow them to reduce other reactive species such as peroxynitrite, peroxynitrite-derived CO(3)(*-), peroxyl radical, and less efficiently H(2)O(2). By doing so, SOD mimics can decrease both primary and secondary oxidative events, the latter arising from the inhibition of cellular transcriptional activity [121]. Also the selenoorganic compound ebselen, 2-phenyl-1,2-benzisoselenazol-3(2H)-one, exhibits activity as an enzyme mimic. In the presence of thiols, ebselen mimics the catalytic activities of phospholipid hydroperoxide glutathione peroxidase. The reaction catalyzed is that of a glutathione (GSH) peroxidase (i.e., the reduction of a hydroperoxide at the expense of thiol). Ebselen also has properties such as free radical and singlet oxygen quenching [122]. Filipovska et al. [123] prepared the synthesis and characterised a triphenylphosphonium-conjugated peroxidase mimetic (2-[4-(4-triphenylphosphoniobutoxy) phenyl]-1,2-benzisoselenazol)-3(2H)-one iodide (MitoPeroxidase)), which contains an ebselen moiety covalently linked to a triphenylphosphonium (TPP) cation. MitoPeroxidase and ebselen were effective antioxidants that degraded phospholipid hydroperoxides, prevented lipid peroxidation, and protected mitochondria from oxidative damage. Both MitoPeroxidase and ebselen decreased apoptosis induced by oxidative stress, suggesting that they can decrease mitochondrial oxidative stress.

In a similar way, the “xenohormesis hypothesis” [124] suggests that many of the health benefits of dietary phytochemicals, particularly those of secondary metabolites produced by plant under stress, may work because they activate an evolutionary ancient mechanism that allows animals and fungi to pick up on chemical stress signals from plants [94]. Under stressful conditions such as cold or drought, when plants ramp up their production of these specialized chemical compounds, by eating the plants, the benefit is transferred to animals [125]. Xenohormesis hypothesis claims that organisms have evolved to respond to stress signaling molecules produced by other species in their environment. In this way, organisms can prepare in advance for a deteriorating environment and/or loss of food supply [124]. The Mediterranean diet is an example of xenohormetic compounds. Plants, such as grape vine and olive trees in the Mediterranean basin, have developed an array of antioxidant defenses to protect themselves from environmental stress [125]. Nevertheless, Sinclair's xenohormesis hypothesis [124] needs additional studies and evidence of support. On the other hand, mitohormesis or mitochondrial hormesis suggests that increased mitochondrial activity and specifically generation of oxidative stress due to this metabolic increase exert positive biological effects ultimately promoting health [126, 127]. Schulz et al. [38] demonstrated that reduced glucose availability promotes the formation of reactive oxygen species (ROS), induces catalase activity, and increases oxidative stress resistance and survival rates of *Caenorhabditis elegans*.

More and more results indicate the possibility of using hormesis as a mild stress in the therapy of human beings (e.g., in longevity and aging research), but much more research in this field will be required. In order to use hormesis to prevent aging and aging-related diseases, according to Le Burg [127], hormesis should not be difficult to practice, be easy to use, inexpensive, and not time-consuming.

### 2.3. Exercise

Any factor that influences the propensity of O_2_ to interact with ubisemiquinone (Q-) will likely increase free-radical production. Exercise involves a large flux of energy and a shift in substrate metabolism in mitochondria from state 4 to state 3. This shift causes an increase in superoxide production [128]. Indeed, a single bout of exercise increases metabolism and oxidative stress during and immediately after exercise [129–131]. While a single bout of exercise of sedentary animals is likely to cause increased detrimental oxidative modification of proteins [132], moderate daily exercise appears to be beneficial by reducing the damage in rat skeletal muscle [133]. Organisms challenged by an oxidative stress often decrease their rate of metabolism, which presumably would lead to a corresponding decrease in their rate of free radical generation [134, 135]. Due to the effect of hormesis, increased O_2_ consumption during sporting activity also increases free radical defense systems by, for example, increasing muscle levels of SOD, glutathione peroxidase and reduced glutathione (GSH) [136]. Consistent with the concept of mitohormesis, exercise-induced oxidative stress ameliorates insulin resistance and causes an adaptive response promoting endogenous antioxidant defense capacity. However, supplementation with antioxidants may preclude these health-promoting effects of exercise in humans [137] by the abrogation of free-radical signaling through antioxidant supplementation which prevents an adaptive response in the mitochondria to increased free radical levels necessary to induce a stress response [138].

A person should thus practice regular, but moderate physical activity. Regular or repeated bouts of exercise are associated with a lower resting metabolic rate, higher antioxidant activity, and lower oxidation of LDLs and more protection against oxidation of proteins and DNA [139, 140]. Additionally, the mechanisms of moderate exercise include improved insulin sensitivity and reductions of oxidative stress and inflammation. Vigorous exercise may also induce moderate heat stress; increasing body temperature due to the increased metabolism and heat production of the skeletal muscle tissue might induce HSPs. Endurance training adaptation causes an increased efficiency in ATP synthesis at the expense of a potential increase in oxidative stress that is likely to be compensated by enhanced activities of antioxidant enzymes [141] and proteasome [133].

### 2.4. Mitochondrial Uncoupling

It has long been known that respiration and mitochondrial ATP synthesis are coupled. Uncoupling is defined as a condition in which the rate of electron transport can no longer be regulated by an intact chemiosmotic gradient. Uncoupling agents dissipate the chemical gradient, usually by creating a mechanism by which protons can escape the intermembrane space (or otherwise “short out” the gradient), allowing an increase in electron transport.

One postulated mechanism to reduce mitochondrial oxidant production is to increase the rate of metabolic uncoupling [142]. Mitochondrial uncoupling has been proposed as a mechanism that reduces reactive oxygen species production and could account for the paradox between longevity and activity [143]. When oxygen consumption is uncoupled from ATP generation, heat is produced. However, the consumption of oxygen without ATP production would also reduce the level of free molecular oxygen potentially available for superoxide anion formation. Small reductions in metabolic flux through the electron transport chain occur at the cost of increased upstream substrate levels [144]. This increased concentration of reduced upstream substrates allows a larger generation of ROS.

When the mitochondria are uncoupled and membrane potential is low, animals might actually produce less free radicals when expending the most energy [25]. This theoretical postulate was confirmed observationally in mice when it was shown that across individuals it was those individuals with the highest energy metabolic rates that lived the longest, and such individuals had greater uncoupling of their muscle mitochondria [145].

Examples of uncoupling agents are the uncoupling proteins (UPCs), a family of transporters belonging to the mitochondrial carrier protein superfamily, which is found in all eukaryotic organisms [146]. An uncoupling protein is a mitochondrial inner membrane protein that can dissipate the proton gradient before it can be used to provide the energy for oxidative phosphorylation [147]. Uncoupling proteins are present in mitochondrial inner membrane and mediate free fatty acid-activated, purine-nucleotide-inhibited H+ reuptake. UCPs can modulate the tightness of coupling between mitochondrial respiration and ATP synthesis [148]. There are at least five types known in mammals: UCP1, also known as thermogenin, UCP2, UCP3, UCP4, and UCP5. Research data show that the general role of the UPC protein family is protection against oxidative stress, since the acceleration of respiration due to UCP-mediated uncoupling would lead to a reduction in ROS production by the respiratory chain [149]. After Ricquier [150], the ancient function of the UCPs may rather be associated with adaptation to oxygen and control of free radicals than to thermogenesis. Also exercise influences the gene expression of UCPs. Uncoupling of oxidation of respiratory substrates and phosphorylation of ADP lowers the efficiency of ATP formation [74]. At the same time, it may diminish the formation of ROS by decreasing the mitochondrial membrane potential and thereby stimulating the electron transport and oxygen consumption. This is due to shortening of the half-life of semiquinone, an intermediate of the electron transport system, which can transfer the electron to molecular oxygen, generating superoxide radicals, [151] and then hydrogen peroxides [152]. It should be mentioned that uncoupling agents could have severe health risks. As such, dinitrophenol (DNP) is a metabolic poison that acts by uncoupling oxidative phosphorylation, leading to increased basal metabolic rates and symptoms like a feeling of warmth, sweating, weight loss, increased heart rate, breathing rate, uncontrolled hyperthermia, and death [153–155]. Concerns about dangerous side effects and rapidly developing cataracts resulted in DNP being discontinued in the United States by the end of 1938 although some more recently beneficial actions of DNP for neurodegenerative diseases and other neurological disorders were reported [156, 157]. It is an illegal weight loss agent that is still used by body builders and is freely available on many Internet websites [155].

### 2.5. Metal Ions Sequestration

Metals such as iron, copper, chromium, vanadium, and cobalt are capable of redox cycling in which a single electron may be accepted or donated by the metal. This action catalyzes reactions that produce reactive radicals and can produce reactive oxygen species. All transition metals, with the exception of copper, contain one electron in their outermost shell and can be considered free radicals. Copper has a full outer shell, but loses and gains electrons very easily making itself a free radical [158]. Iron has the ability to gain and lose electrons (i.e., Fe^2+^ *↔* Fe^3+^) very easily. This property makes iron and copper two common catalysts of oxidation reactions.

It is clear that any increase in the levels of superoxide anion, hydrogen peroxide, or redox-active metal ions is likely to lead to the formation of high levels of hydroxyl radical by the chemical mechanisms outlined above. Therefore, the valence state and bioavailability of redox-active metal ions are key determinants in their ability to participate in the generation of reactive oxygen species. Potential strategies to decrease oxidative stress may involve not only an overall reduction of oxidative stress, but also the use of iron and other metal chelators hampering Fenton-type chemistry [159]. Some compounds contribute to an antioxidant defense by chelating transition metals and preventing them from catalyzing the production of free radicals in the cell. Metal-chelating antioxidants such as transferrin, albumin, and ceruloplasmin avoid radical production by inhibiting the Fenton reaction catalyzed by copper and iron. Particularly important is the ability to sequester iron, which is the function of iron-binding proteins such as transferrin and ferritin [160]. Cells have a not-well-characterized pool of low-molecular-weight iron [161, 162]. If these come into contact with ascorbate, prooxidant effects may occur. Ascorbic acid reduces Fe(III) to Fe(II) which reduces oxygen to hydroxyl radical [158]. According to Herbert [163], for consumer protection, every advertisement and label for vitamin C and/or iron supplements should warn: “do not take this product until your blood iron status has been determined.” Six percent of Americans are in a negative iron balance, and such multivitamin/mineral product may help them. Twelve percent of Americans are in positive iron balance and such product may hurt them.

### 2.6. Lifestyle Approaches to Reduce Oxidative Stress

Variety of lifestyle factors have been found that are oxidative stress generators, as well as those that reduce this condition. Some of them are briefly discussed.

Consumption of vegetables and plant-derived foods and beverages has positive effect on the prevention of age associated diseases like coronary heart disease and atherosclerosis as well as for longevity [164–166]. Based on the analysis of available epidemiological data, a daily intake of at least 400 g of fruits and vegetables has been recommended by the World Health Organisation [167]. Avoiding calorie-dense refined sugars, saturated fats, and processed foods and replacing them with nutrient-dense but calorie-poor vegetables, fruits, and legumes will result in a vastly increased intake of health-enhancing phytonutrients, including key vitamins and minerals, antioxidants, flavonoids, and other still undiscovered compounds important for our cells to work properly. While there is no formal recommendation for the amount of antioxidants we need [41], the best way to obtain antioxidants is from a varied diet. Foods rich in antioxidants include all fresh and seasonal fruit and vegetables (peppers, apples, onions, pineapple, dark leafy vegetables, flaxseeds, walnuts, pumpkin seeds, and olives), olive oil, and fish. Also omega-3 fatty acids EPA and DHA appear to stabilize mitochondrial membranes [168–170] as well as magnesium which is required for the oxidation-reduction steps in mitochondria since magnesium deficiency enhances free radical formation [171, 172]. Membrane lipids increase their sensitivity to oxidative damage as a function of their unsaturation. Pamplona et al. [173, 174] analyzed fatty acids of liver mitochondria from eight mammals and showed that the total number of double bonds and the peroxidizability index is negatively correlated with maximum life span. Authors proposed that, during evolution, a low degree of fatty acid unsaturation in liver mitochondria may have been selected in longevous mammals in order to protect the tissues against oxidative damage, while maintaining an appropriate environment for membrane function.

#### 2.6.1. Eating, Postprandial Oxidative Stress, and Electron Transport in Mitochondria

Nutritional (or dietary) oxidative stress describes an imbalance between the prooxidant load and the antioxidant defense as a consequence of excess oxidative load or of an inadequate supply of the organism with nutrients [175]. In Western societies, a significant part of the day is spent in the postprandial state. Postprandial (*postprandial* means after eating a meal, while preprandial is before a meal) oxidative stress is characterized by an increased susceptibility of the organism toward oxidative damage after consumption of a meal rich in lipids and/or carbohydrates [176]. The generation of excess superoxide due to abundant energy substrate after the meal may be a predominate factor resulting in oxidative stress and a decrease in nitric oxide, which is important to endothelial function. The “three meals a day plus snacks” diet is typical of many modern industrialized countries. In contrast to the continuous availability of food that we currently enjoy, our ancestors were often forced to endure extended periods from many hours to days without food [42]. When overeating, high amounts of energy become available and mitochondria do not operate very efficiently and generate more superoxide. The electron transport always proceeds as fast as energy is removed from the gradient. Electron transport is *driven* by the free energy that is available from the energy carriers, in turn obtained from substrates such as glutamate or Krebs intermediates. It is *restricted* by the chemiosmotic gradient. Anything that increases the turnover of energy from the gradient increases the rate of electron transport proportionally. ROS production thus plummets when ATP demand is high. Decreased proton concentration or membrane potential brings about a nonlinear and a striking decrease in the level of inadvertent electron leaks, and this likely accounts for ROS decrease [177].

The postprandial increase in plasma lipid hydroperoxide content and oxidative stress is restricted when a meal containing oxidized and oxidizable lipids is consumed together with red wine or with procyanidins [178]. Procyanidins can be found in many plants; important sources are red wine, in particular; grape seeds and grape skin, cocoa, pine bark, and green tea. 

A mixture of antioxidant compounds are required to provide protection from the oxidative effects of postprandial fats and sugars. No specific antioxidant can be claimed to be the most important because human food consumption varies enormously. However, a variety of polyphenolic compounds derived from plants appear to be effective dietary antioxidants, especially when consumed with high-fat meals [175].

Besides, glucose in the blood increases after consuming a meal, and for this reason, work instead of resting should be performed in order to maintain an appropriate electron flow and avoid electron leaks. The regulation of energy by the body's circadian rhythms may play a significant role in controlling oxidative stress. In modern society, we are faced with excessive psychological stress, as well as an epidemic of overeating, and the two together appear to have synergistic effects according to Epel [179].

#### 2.6.2. Excessive Psychophysical Stressful Situations

In this paragraph, the question of whether emotional stress contributes to increased oxidative stress will be attempted to be answered. Psychological stress is becoming common in modern society and is known to predispose individuals to several diseases, since stress can diminish the effectiveness of the immune system [180] and possibly also the effectiveness of the antioxidant system and repair processes. It is known that adrenalin is released into the blood when a person faces a stressful situation. If adrenalin is not used for either fight or flight (as it was in the past), it oxidizes in the presence of O_2_ or promotes redox cycling to form superoxide [180] and increases oxidative stress. Emotional stress in humans associates with higher biomarkers for oxidative stress. Emotional stress increases catecholamine metabolism, which increases oxidative stress by increasing the production of free radicals. Similarly, academic stress (stress due to academic commitments, financial pressures, and lack of time management skills among students) [181] or sleep deprivation [182] associate with lower protective antioxidant levels in blood. Eskiocak et al. [181] demonstrated that glutathione and free sulphydryl levels in seminal plasma decreased in subjects undergoing examination stress. Everson et al. [182] found out that recovery sleep normalized antioxidant content in liver and enhanced enzymatic antioxidant activities in both the liver and the heart. Results of their study link uncompensated oxidative stress to health effects induced by sleep deprivation and provide evidence that restoration of antioxidant balance is a property of recovery sleep. Excessive psychophysical stress should be avoided, and we should take time to relax and enjoy the things we like doing.

#### 2.6.3. Sleeping and Melatonin Production

In all organisms, melatonin is produced primarily during the daily period of darkness, with only small amounts being synthesized during the day. For this reason, people should sleep long enough and in complete darkness in order to produce necessary amounts of the endogenous powerful antioxidant melatonin [183, 184] and benefit from its particular role in the protection of nuclear and mitochondrial DNA [185]. Melatonin is an antioxidant that can easily cross cell membranes and the blood-brain barrier [184]. Melatonin is a direct scavenger of OH, O_2_^−^, and NO [185, 186]. Unlike other antioxidants, melatonin does not undergo redox cycling, the ability of a molecule to undergo reduction and oxidation repeatedly.

### 2.7. CoQ10 and Other “New” Compounds

Increased oxidative stress with age may be in part due to a decline in the levels of the endogenous cellular antioxidants, among them also coenzyme Q10 declines significantly with age [187, 188]. Coenzyme Q10 is a naturally occurring antioxidant and a prominent component of mitochondrial electron transport chain. The study of Prahl et al. [189] shows significant age-dependent differences in mitochondrial function of keratinocytes isolated from skin biopsies of young and old donors. From the data, researchers postulate that energy metabolism shifts to a predominantly nonmitochondrial pathway and is therefore functionally anaerobic with advancing age. Coenzyme Q is recognized as an obligatory co-factor for the function of uncoupling proteins and a modulator of the transition pore. Furthermore, data reveal that CoQ10 affects expression of genes involved in human cell signaling, metabolism, and transport and some of the effects of exogenously administered CoQ10 may be due to this property [190]. It was observed also the influence of CoQ10 on endogenous antioxidant defense by increasing SOD2 and GPx [191].

There are some exciting results of the studies on specific new compounds. Recent targeted delivery of antioxidants to mitochondria (that are currently under development) have moved closer to providing protection against mitochondrial oxidative damage. Some encouraging results to prevent senescence were obtained by triphenylphosphonium-conjugated antioxidants (e.g., plastoquinone (SkQ1), MitoQ, and others). Plastoquinone is a very effective electron carrier and antioxidant of chloroplasts and was conjugated with decyltriphenylphosphonium to obtain a cation (called SkQ1) which easily penetrates through membranes. Very low (nano- and subnanomolar) concentrations of 10-(6′-plastoquinonyl) decyltriphenylphosphonium (SkQ1) were found to prolong the lifespan of a fungus (*Podospora anserina*), a crustacean (*Ceriodaphnia affinis*), an insect (*Drosophila melanogaster*), and a mammal (mouse) [192, 193]. These SS peptides scavenge hydrogen peroxide and peroxynitrite and inhibit lipid peroxidation. By reducing mitochondrial ROS, they inhibit mitochondrial permeability transition and cytochrome c release, thus preventing oxidant-induced cell death [194]. More studies, especially long-term studies, are needed to evaluate the effect of a targeted delivery of antioxidants to mitochondria and their role in lifespan and oxidative stress reduction.

There are many other encouraging compounds relevant to this topic, but more detailed discussion exceeds the scope of this paper.

## 3. Conclusion and Perspectives

It is believed that the oxidative damage is the major senescence process, and the mitochondria are the major source of endogenous reactive oxygen species formation while UVR, air pollution, and smoking are the main exogenous sources. Overall, antioxidant defense seems to be in approximate balance with oxygen-derived species generation* in vivo*. There appears to be no great reserve of antioxidant defenses in mammals, perhaps because some oxygen-derived species perform useful metabolite roles. Certain compounds or strategies cause an activation of mitochondrial oxygen consumption and promote increased formation of ROS formation. These serve as molecular signals to exert downstream effects to ultimately induce endogenous defense mechanisms culminating in increased stress resistance and longevity [98]. It also seems that by additional intake of synthetic antioxidants, total antioxidant activity in the blood/cells cannot be further increased or it cannot offer additional protection if one is having an optimal age, nutrition, and lifestyle. Epidemiological studies on synthetic antioxidants failed to completely confirm the beneficial intake of these compounds into our diet. A healthy adult person thus should not need additional vitamin and mineral supplements if he eats varied and diverse foods with a sufficient energy intake. Nevertheless, during aging the oxidative stress of the organism is increasing and approaches to lower the increased ROS formation in our cells should be implemented.

The most efficient preventive step to avoid exogenous free-radical exposure would be to avoid, as much as possible, exposure to endogenous and exogenous ROS generating compounds, including oxygen species, cigarette smoke, and UVR. But since this is not always possible, protection could be obtained by adequate antioxidant protection, decreasing the formation of free radicals, or increasing damage-repair systems of the cells. Approaches for reducing the generation of oxidative stress might be important in aging and disease risk prevention, especially in the second part of our life, when some of the endogenous antioxidant defense systems fail to offer appropriate protection against elevated oxidative stress.

Paradoxically, the efficiency of defense and repair may be enhanced also following exposure to ROS since expression of many DNA repair enzymes is upregulated following oxidative stress [38, 195, 196]. There are, however, promising studies in the field of antioxidant mimetics and investigation of the effects of CR and hormesis. However, also here we should wait for some more conclusive results from these fields [197]. To date, only caloric restriction (with adequate vitamin and mineral intake) has obtained scientifically based results for the prolonging of life. It could be concluded that prevention of mitochondrial ROS generation is a more efficient approach to decreasing oxidative stress (e.g., by CR) than quenching any already formed free radicals with antioxidants, since the lifetime of most aggressive free radicals is very short (e.g., OH^*∙*^) and they react with the first compound encountered (e.g., protein, DNA).

In general, alterations of electron flow cause electron leaks from the complexes and consequently lead to the generation of more ROS. On the other hand, the reduction of energy metabolism may actually reduce ROS generation from mitochondria and consequently extend lifespan [198]. In either case, avoiding electron leakage from electron transport and the resultant ROS production seem to be essential for a normal life. In order to reduce endogenous oxidative stress levels and its damage, research should focus also in some reasonable nutritional and lifestyle approaches mentioned in this paper. Further studies are needed to find new mild stresses or before hormesis of known hermetic agents becomes used also in human beings. Integration of alternative approaches to decrease oxidative stress is still at its beginning. There is a need for a general explanation of causes for oxidative stress reduction and for this reason the aim of this paper is to stimulate the scientific discussion and further research into this matter. 
